# Generation of pig induced pluripotent stem cells using an extended pluripotent stem cell culture system

**DOI:** 10.1186/s13287-019-1303-0

**Published:** 2019-06-27

**Authors:** Junjun Xu, Leqian Yu, Jianxiong Guo, Jinzhu Xiang, Zheng Zheng, Dengfeng Gao, Bingbo Shi, Haiyang Hao, Deling Jiao, Liang Zhong, Yu Wang, Jun Wu, Hongjiang Wei, Jianyong Han

**Affiliations:** 1 State Key Laboratory for Agrobiotechnology, College of Biological Sciences, China Agricultural University, Beijing, 100193 China; 2 Department of Molecular Biology, University of Texas Southwestern Medical Center, Dallas, TX 75390 USA; 3 Hamon Center for Regenerative Science and Medicine, University of Texas Southwestern Medical Center, Dallas, TX 75390 USA; 4 Key Laboratory of Animal Gene Editing and Animal Cloning in Yunnan Province, Kunming, 650201 China; 5 College of Biological Sciences, China Agricultural University, Beijing, 100193 China; 6 CAS Key Laboratory of Pathogenic Microbiology and Immunology, Institute of Microbiology, Chinese Academy of Sciences, Beijing, 100101 China; 7 State Key Laboratory of Stem Cell and Reproductive Biology, Institute of Zoology, Chinese Academy of Sciences, Beijing, 100101 China; 8 College of Veterinary Medicine, Yunnan Agriculture University, Kunming, 650201 China

**Keywords:** Pig iPS cells, Extended pluripotency, Chimera, EPS cells

## Abstract

**Background:**

Pigs have emerged as one of the most popular large animal models in biomedical research, which in many cases is considered as a superior choice over rodent models. In addition, transplantation studies using pig pluripotent stem (PS) cell derivatives may serve as a testbed for safety and efficacy prior to human trials. Recently, it has been shown that mouse and human PS cells cultured in LCDM (recombinant human LIF, CHIR 99021, (S)-(+)-dimethindene maleate, minocycline hydrochloride) medium exhibited extended developmental potential (designated as extended pluripotent stem cells, or EPS cells), which could generate both embryonic and extraembryonic tissues in chimeric mouse conceptus. Whether stable pig induced pluripotent stem (iPS) cells can be generated in LCDM medium and their chimeric competency remains unknown.

**Methods:**

iPS cells were generated by infecting pig pericytes (PC) and embryonic fibroblasts (PEFs) with a retroviral vector encoding Oct4, Sox2, Klf4, and cMyc reprogramming factors and subsequently cultured in a modified LCDM medium. The pluripotency of PC-iPS and PEF-iPS cells was characterized by examining the expression of pluripotency-related transcription factors and surface markers, transcriptome analysis, and in vitro and in vivo differentiation capabilities. Chimeric contribution of PC-iPS cells to mouse and pig conceptus was also evaluated with fluorescence microscopy, flow cytometry, and PCR analysis.

**Results:**

In this study, using a modified version of the LCDM medium, we successfully generated iPS cells from both PCs and PEFs. Both PC-iPS and PEF-iPS cells maintained the stable “dome-shaped” morphology and genome stability after long-term culture. The immunocytochemistry analyses revealed that both PC-iPS and PEF-iPS cells expressed OCT4, SOX2, and SALL4, but only PC-iPS cells expressed NANOG and TRA-1-81 (faint). PC-iPS and PEF-iPS cells could be differentiated into cell derivatives of all three primary germ layers in vitro. The transcriptome analysis showed that PEF-iPS and PC-iPS cells clustered with pig ICM, Heatmap and volcano plot showed that there were 1475 differentially expressed genes (DEGs) between PC-iPS and PEF-iPS cells (adjusted *p* value < 0.1), and the numbers of upregulated genes and downregulated genes in PC-iPS cells were 755 and 720, respectively. Upregulated genes were enriched with GO terms including regulation of stem cell differentiation, proliferation, development, and maintenance. And KEGG pathway enrichment in upregulated genes revealed *Wnt*, *Jak-STAT*, *TGF-β*, *P53*, and *MAPK* stem cell signaling pathways. Fluorescence microscopy and genomic PCR analyses using pig mtDNA-specific and GFP primers showed that the PC-iPS cell derivatives could be detected in both mouse and pig pre-implantation blastocysts and post-implantation conceptuses. Quantitative analysis via flow cytometry revealed that the chimeric contribution of pig PC-iPS cells in mouse conceptus was up to 0.04%.

**Conclusions:**

Our findings demonstrate that stable iPS cells could be generated in LCDM medium, which could give rise to both embryonic and extraembryonic cells in vivo. However, the efficiency and level of chimeric contribution of pig LCDM-iPS cells were found low.

**Electronic supplementary material:**

The online version of this article (10.1186/s13287-019-1303-0) contains supplementary material, which is available to authorized users.

## Background

Human induced pluripotent stem (iPS) cells hold great therapeutic promise for regenerative medicine. Human iPS cells can proliferate indefinitely in culture and differentiate into all cell types in an adult body, thereby providing unlimited source material for cell-based therapies to treat numerous disorders. Before translating to the clinic, however, it is imperative to test the safety and efficacy of iPS cell-based therapies using animal models. Owing to easy accessibility, low costs, and a range of available genetic and molecular tools, rodents, in particular mice, have been the most popular animal model for pre-clinical trials. However, in many instances, rodent models cannot accurately reflect the human conditions due to significant differences in development and physiology [1]. Pigs are more similar to humans than the rodents in organ size, physiology, and anatomy, and thus autologous and/or homologous transplantation using pig iPS cell derivatives represents a superior model for regenerative medicine. Moreover, with the development of interspecies blastocyst complementation, human organs generated in pigs may help solve the worldwide shortage of human organs for transplantation in the future [2–4]*.* In this regard, chimeric-competent pig iPS cells can serve as a con-species control, providing invaluable information on the molecular and functional features of derived organs. Despite the potential, however, authentic pig embryonic stem (ES) cells have yet been established, and the maintenance of pig iPS cells still depends on ectopic expression of exogenous reprogramming factors. Importantly, neither pig ES nor iPS cells so far have met the gold standard functional assay for pluripotency: germline-competent chimeras. Therefore, improving the quality of pig iPS cells is of major importance for future pig PS cell-based pre-clinical studies.

Rodent studies have demonstrated that PS cells at least exist in two distinct pluripotent states in culture: naïve and primed, which differ in their molecular features and chimeric potential. Mouse ES cells represent the naïve pluripotent state and can produce germline chimeras [5, 6]*.* In contrast, The primed mouse epiblast stem cells (EpiSCs) inefficiently contributed to chimera formation following blastocyst injection [3, 7, 8]. Mouse ES cells can be maintained in a ground state culture condition (2iL: GSK3 and Mek1/2 inhibitors, plus LIF), while mouse EpiSCs are typically cultured in bFGF and Activin-A containing medium [9]. Although pig naïve-like and primed iPS cells have been generated using different culture media, different reprogramming factor combinations, and different starting cell types [10–14] and transgene-free intermediate type piPS have been also generated [15], germline-competent pig iPS cells are still not available. Recently, it has been shown that a novel PS cell type, termed extended pluripotent stem (EPS) cells cultured in the LCDM medium, can be generated from both mouse and human somatic cells, ES cells, and blastocysts. These EPS cells show superior chimeric competency to both embryonic and extraembryonic lineages in mice than any other PSC types [16]*.* To date, LCDM culture has not been tested for the generation of pig iPS cells, and if so, whether they can contribute to chimeras remains unknown.

Besides the culture condition, another factor likely influencing the outcome of iPS cell generation is the starting cell types. It is known that different starting cell types share distinct transcriptional features during iPS cell generation and, as a result, may exhibit different reprogramming dynamics and efficiency and yield iPS cells with different quality [17]*.* For example, reprogramming efficiencies of mouse adipose stem cell and neural stem cell were higher than that of mouse embryonic fibroblasts (MEFs) [18]. Pericytes, the microvascular mural cells, are abundant in the body and can differentiate into multiple cell types of the mesenchymal lineage, suggesting higher cellular plasticity than that of fibroblasts [19, 20]. Therefore, pericytes may represent superior starting cells for pig iPS cell generation. In this study, we generated both PC- and PEF-derived iPS cells (named PC-iPS and PEF-iPS, respectively) in a modified LCDM medium and characterized the pluripotency of established PC-iPS cells both in vitro and in vivo.

## Methods

### Derivation and culture of pig embryonic fibroblasts (PEFs) and meninges microvascular pericyte cells (PCs)

To derive pig embryonic fibroblasts (PEFs), whole uteruses of pregnant female Nongda xiang pigs at embryonic day 40 (E40) were dissected and the fetuses were surgically separated. Head, limbs, and internal organs were removed under sterile conditions, and the remaining tissue was digested with 0.5% collagenase IV (17104-019; Gibco) and plated in a 6-well plate (703,001; NEST). PEFs were cultured in DMEM (11,960; Gibco) with 10% FBS (SE200-ES; VISTECH) under 5% CO_2_ at 37 °C. To derive microvascular pericyte cells (PCs), the pig meninges were isolated from the pig fetus heads by ophthalmic forceps under a stereoscopic microscope and digested by 0.5% Collagenase IV at 37 °C, on a shaking plate (IKA, KS130) for 2 h. Microvascular vessels were collected through a 40-mesh sieve (431,750; Corning), and large blood vessels were removed through sedimentation. The obtained microvascular tissues were cultured in 6-well plates in pericyte culture medium (1201; ScienCell) under 5% CO_2_ at 37 °C.

### Generation of pig induced pluripotent stem cells from PCs (PC-iPS) and PEFs (PEF-iPS)

When reaching 70% confluence, PCs (P3) and PEFs (P3) were infected with a retroviral vector (RTV-010, Cell Biolabs, Inc.) encoding pig Oct4, Sox2, Klf4, and cMyc. One day later, the medium containing retrovirus was replaced with fresh medium (DMEM containing 10% FBS). On day 3 post-infection, the cells were passaged as single cells onto mitotically inactivated mouse embryonic fibroblasts (MEFs). On day 5, the medium was replaced by 50% (*v*/*v*) mTeSR1™ (base medium 85,851 plus supplement 85,852; Stem Cell Technologies) and 50% (*v*/*v*) modified LCDM medium [16]. It was reported that the addition of vitamin C improved the reprogramming efficiency and expression of pluripotency genes in iPS cells [21]. Thus, vitamin C (A4544; Sigma) was added to the LCDM medium (designated as LCDMV medium). LCDMV medium contains 50% (*v*/*v*) Neurobasal™ Medium (21103-049; Gibco), 50% (*v*/*v*) DMEM/F12 (10565-018; Gibco), 1×N2 (17502-048; Gibco), 0.5× B27 (12587-010; Gibco), 5% KOSR (10828-028; Gibco), LIF (10 ng/ml, 300-05-1000; Peprotech), CHIR99021 (1 μM, 4423; Tocris,), (S)-(+)-dimethindene maleate (2 μM, 1425; Tocris), minocycline hydrochloride (2 μM, sc-203,339; Santa Cruz), and vitamin C (40 μg/ml, A92902; Sigma). On day 16, cell colonies were individually picked and plated onto newly prepared MEFs in 12-well plates (712,001; NEST). After colonies had attached and expanded, the medium was changed to LCDMV only. We observed that between passage 1 (P1) to P4, the morphology of PC-iPS and PEF-iPS cell colonies changed from flat to “dome” shaped. The PC-iPS and PEF-iPS cells were passaged as single cells using StemPro™ Accutase™ Cell Dissociation Reagent (A1110501; Gibco) and cryopreserved in LCDMV containing 10% DMSO (D2650; Sigma).To fluorescently label PC-iPS cells, in brief, 7.5 μl Lipofectamine®3000 reagent (L3000015; Invitrogen) was diluted with 250 μl Opti-MEM® (31,985,070; Gibco); 3 μg pB513B-1 (System Biosciences, CA, USA) and 1 μg PCAGPBase plasmids [22] were also diluted with 250 μl Opti-MEM® and mixed with 10 μl P3000™ reagent (L3000015; Invitrogen). Next, diluted plasmids were added to the diluted Lipofectamine® 3000 reagent and incubated at room temperature for 15 min. After incubation, the transfection mixture was added into one well of a 6-well plate containing 5 × 10^5^–1 × 10^6^ cells. PC-iPS cells were cultured for an additional 48 h in the presence of the transfection mixture before changing to new LCDMV medium. The expression of green fluorescent protein (GFP) in PC-iPS cells was confirmed by observing under a fluorescence microscope (Olympus ZX71), and GFP+ cells were sorted by flow cytometry (Beckman MoFlo XDP).

### Alkaline phosphatase (AP) staining

PC-iPS (P12) and PEF-iPS (P12) cells were fixed with 4% paraformaldehyde (3053589-4, Sangon Biotech) at room temperature for 5 min followed by washing twice with PBS. Alkaline phosphatase (AP) staining solution was prepared by mixing Fast Red Violet (FRV) solution, naphthol AS-BI phosphate solution, and deionized water at 2:1:1(*v*/*v*/*v*) ratio. One-milliliter AP staining solution was added to one well of a 6-well plate containing fixed iPS cells and incubated for 15 min at 37 °C followed by PBS washing. Two hundred-milliliter Fast Red Violet (FRV) solution contains 16 ml 0.1 μM Sodium Nitrite (237,213, Sigma) + 16 ml 0.4 μM HCl (SCRC) + 80 mg Fast Red Violet LB Base (274,054, Sigma-Aldrich) + 168 ml deionized water; 100 ml naphthol AS-BI phosphate solution contains 50 ml 0.1 μM 2-amino-2-methyl-1,3-propanediol (pH 9.5) (A9754, Sigma-Aldrich) + 200 mg naphthol AS-B2 (N2125, Sigma-Aldrich) + 50 ml deionized water.

### Embryoid body formation and in vitro differentiation

Embryoid bodies (EBs) of PC-iPS (P12) and PEF-iPS (P12) cells were generated in the LCDMV culture medium on a shaking plate (IKA KS130) at 37 °C under 5%.

CO_2_ for 3–6 days and collected by natural sedimentation. The EB spheres were plated into a 12-well plate (712,001, Nest), and the medium was replaced every 2 days with fresh DMEM containing 10% FBS. After 7–10 days, iPS cell differentiation was analyzed by immunocytochemistry.

### Karyotype analysis

PC-iPS (P28) and PEF-iPS (P18) cells were passaged and cultured for 2 days, then the culture medium was replaced with 2 ml of fresh LCDMV medium with 200 μl KaryoMAX Colcemid Solution (15210-040; Gibco) in one 6-well, and cells were incubated at 37 °C under 5% CO_2_ for 2.5 h. The PC-iPS and PEF-iPS cells were then collected and resuspended in 5 ml 0.075 M KCl (SCRC). After incubation for 30 min at 37 °C, 0.5 ml of fixative (methanol (SCRC) and acetic acid (SCRC) at 3:1 (*v*/*v*) ratio) was added to the KCl solution and centrifuged at 1000 rpm for 10 min at 4 °C. The supernatant was then removed, and 10 ml of ice-cold fixative was added. The cells were incubated on ice for 30 min, and the fixation step was then repeated but with on-ice incubation for 1 h. The final cell precipitation comprising a single-cell suspension was dropped on to microscope slides, incubated, treated with 0.01% Trypsin-EDTA, stained with the Rapid Giemsa Staining kit (E6073141; BBI Life Science), and photographed (Leica; DFC365 FX).

### Immunohistochemistry

The PCs (P4), PC-iPS (P12), and PEF-iPS (P12) cells were fixed with 4% paraformaldehyde (3053589-4, Sangon Biotech) at room temperature for 20 min and then washed twice in DPBS (C14190500BT; Gibco). Cells were then treated with 0.5% Triton X 100 solution (0694; Amresco) for 50 min and washed three times with 0.1% Triton X 100. Blocking solution (P0102; Beyotime) was then applied for 1 h at 26 °C. Primary antibodies were then added, and the plates containing cells were incubated overnight at 4 °C. The next day, plates were washed three times with 0.1% Triton X-100 on a shaking plate at 26 °C to remove the primary antibody. A secondary antibody was then added to the plates which were incubated at 26 °C for 2 h. Plates were then washed twice on the shaking plate at 26 °C to remove the secondary antibody, DAPI (1:5000) diluted with DPBS was then added, and fluorescence signals were detected by a fluorescence microscope*.* NG2 (sc-53,389; Santa Cruz) (1:200) and α-SMA (ab119952; Abcam) (1:250) were used for characterization of PCs; Nanog (500-P236; Peprotech) (1:250), Sox2(SC365823; Santa Cruz) (1:250), Oct4 (SC8826; Santa Cruz) (1:200), Tra-1-81 (ab16289; Abcam) (1:200), and Sall4 (GTX109983; GenTex) (1:200) were used for characterization of pig iPS cells; α-SMA (ab5694; Abcam) (1:250), vimentin (ab92547; Abcam) (1:250), and β-tubulin (ab18207; Abcam) (1:250) were used for checking differentiation of pig iPS EBs into three germ layers. Primary antibodies were diluted in primary antibody solution (P0103, Beyotime). Donkey anti-mouse IgG (H+L) highly cross-adsorbed secondary antibody (1:750) (Alexa Fluor 594; A21203, Thermo Fisher Scientific) and Donkey anti-Rabbit IgG (H+L) highly cross-adsorbed secondary antibody (1:750) (Alexa Fluor 594; A21207, Thermo Fisher Scientific) were used as secondary antibodies. Secondary antibodies were diluted in secondary antibody solution (P0108, Beyotime).

### DNA and RNA extraction and PCR/RT-PCR

DNA and RNA were extracted using extraction kits (DP304-03 and DP430, respectively; Tiangen), in accordance with the manufacturer’s instructions*.* Primers were designed online (https://www.ncbi.nlm.nih.gov/tools/primer-blast/) and synthesized by the Beijing Genomics Institute (Beijing, China). The list of primers used in this study is provided in Additional file 1: Table S1*.* Polymerase chain reaction, nested PCR, and RT-PCR programs were conducted using PrimeSTAR®GXL DNA Polymerase (R050A; Takara) per the manufacturer’s recommendations.

### Alignment of RNA-seq data and differential expression analysis

Two micrograms of total RNA obtained from PC-iPS and PEF-iPS cells was shipped on dry ice to Anoroad Gene Technology Corporation (Beijing, China) for RNA sequencing. The low-quality reads and adaptor sequences were trimmed with Trimmomatic [23]. Clean reads were aligned to pig genome Ssc11.1 (from Ensembl) by Hisat2 [24]. Gene counts were calculated by counting the overlap of reads on each gene with HT-seq [25], and the expression level was normalized as RPKM with gene annotation file from Ensembl (release 94) and edgeR package in R [26]. Differential expression genes were identified by DESeq2 package, and functional enrichment for Gene Ontology (GO) and KEGG were performing with GOstats package [27]. Pig embryo RNA-seq data were from NCBI Sequence Read Archive (SRA326708), including 16 samples of porcine oocytes and in vivo embryos [28].

### Mouse embryo collection, culture, and transplantation

Mouse embryos were collected and cultured according to the following procedures: on day 1, pregnant mare’s serum gonadotropin PMSG (100 μl, 10 U; Ningbo Second Hormone Factor) was injected into the abdominal cavity of mice. On day 3, mice were injected with hCG (100 μl, 10 U; Ningbo Second Hormone Factor) and caged with a male mouse. On day 4, mice bearing a vaginal plug were isolated to produce E0.5 pregnant mice. The other female mice were caged with a castrated male mouse to prepare E2.5 pseudopregnant mice. On day 5, the E1.5 pregnant mice were sacrificed, and 2-cell embryos were flushed out with EmbryoMax® M2 Medium (MR-015-D; Millipore) and cultured in EmbryoMax® KSOM Mouse Embryo Media (MR-121-D; Millipore) under atmospheric conditions of 5% CO_2_ and 37 °C. On day 6, GFP-labeled PC-iPS cells were injected into 4- to 8-cell embryos by microinjection and the resulting embryos were cultured in KSOM as before. For blastocyst injection, on day 7, early blastocysts were flushed out with M2 medium and GFP PC-iPS cells were microinjected into the blastocysts. Chimeric blastocysts derived from 4 to 8 cells, and blastocyst injections were then transplanted into the uterine horns of mice.

### Microinjection of GFP PC-iPS cells into 4- to 8-cell embryos and early blastocysts

GFP-piPS cells were cultured in LCDMV medium for 2 days, then dissociated with accutase for 3–5 min. Cells were then collected into 1.5-ml tubes, washed with PBS, and then resuspended in M2 medium. Five to ten GFP-piPS cells were injected into the 4- to 8-cell embryos and blastocysts which were then cultured for 36 h and 6 h, respectively, prior to transplantation into the uterine horns of surrogate females.

### Immunohistochemistry of late-stage blastocysts

After the injected 4- to 8-cell embryos developed into blastocysts, some of the late blastocysts were used for immunohistochemistry. CDX2 (MU392A-UC; Biolegend) was used as a TE marker, Nanog was used as a pluripotent marker, and anti-GFP antibody-ChIP Grade (ab290; Abcam) was used as a marker for GFP PC-iPS cells.

### Detection of GFP fluorescence in mice fetuses and extraembryonic tissues

Pregnant female mice between embryonic days 10.5 and 13.5 (E10.5–13.5) were sacrificed by cervical dislocation, and their uteruses were surgically removed. Fetuses and extraembryonic tissues (placentas, fetal membrane, and placenta implantations) were separated manually under a fluorescence stereoscope (Olympus SZX16). Chimeric contribution of piPS cells was examined by a combination of fluorescence stereoscopic microscopy, flow cytometry, and PCR.

### Generation of chimeric pig embryos and embryo transfer

#### Pig oocytes collection and culture

Pig ovaries were obtained from a Hongteng slaughterhouse (Kunming, Yunnan, China) and were maintained at 25–30 °C during transportation to the laboratory. The selection standard for ovarian follicles includes a diameter of between 3 and 6 mm and cumulus-oocyte complexes (COCs) comprising of more than 3 layers of cumulus cells, in accordance with published recommendations [29, 30]. Oocytes were cultured in vitro maturation medium (IVM) at 38.5 °C under 5% CO_2_. After maturation of oocytes, cumulus cells were removed by addition of 0.1% (*w*/*v*) hyaluronidase (H4272, Sigma).

#### Generation and culture of pig embryos by somatic nuclear transfer (SCNT)

Recipient pigs were raised at the Animal Center of Yunnan Agricultural University (Yunnan, China). Donor cells from embryonic fibroblasts (Yorkshire-01) obtained from Yorkshire pigs were thawed and cultured in DMEM (C11995500BT; Gibco) containing 10% FBS (VS500T; Ausbian) at 38.5 °C under 5% CO_2_. SCNT was performed according to the published procedures [31]. Briefly, enucleation fibroblasts and donor cell nuclear transfer were conducted using a micromanipulation system (Narishige; NT-88-v3), and SCNT embryos were subsequently transferred to porcine zygote medium-3 (PZM-3) until microinjection.

#### Microinjection of GFP PC-iPS cells into pig 4- to 8-cell embryos

Ten to 15 GFP-labeled PC-iPS cells were injected into the embryos between 4 to 8 cells and compaction stage. The chimeric embryos were then cultured in PZM-3 medium for 6–30 h, and blastocysts were counted before transplantation into recipient pigs.

#### Detection of GFP fluorescence in pig fetuses and extraembryonic tissues

The chimeric blastocysts were transferred into the tip of a uterine horn of recipient pigs weighing between 100 and 120 kg. Pregnancies were detected by B ultrasounds from day 16 of embryonic development post-transfer. Fetuses were collected between E24 and E30, and fluorescence signals were detected by stereoscopic microscopy (Nikon; SMZ18) and chimeric contribution of GFP PC-iPS cells was confirmed by PCR.

## Results

### Generation of PC-iPS and PEF-iPS cells in a modified LCDM medium

**Fig. 1:**
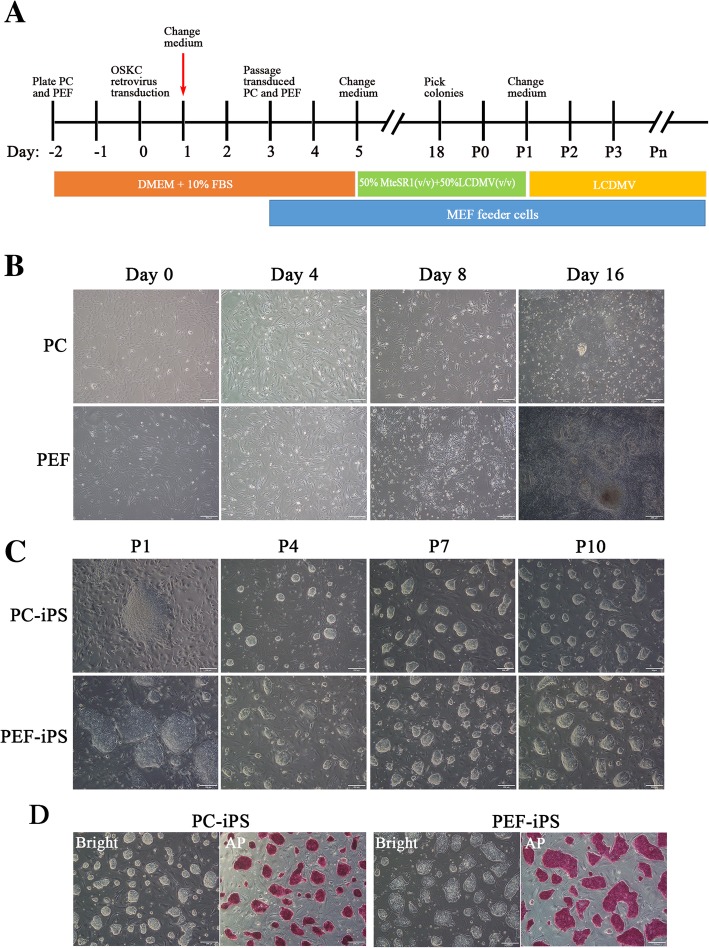
Generation of PC-iPS and PEF-iPS**. a** A schematic illustration of the generation of Pig iPS. **b** The process of generation of PC-iPS and PEF-iPS, scale bar 200 μm. **c** Colony morphologies of PC-iPS and PEF-iPS cells changed from P1 to P4 and maintained stable dome-shaped from P4 to P10, scale bar 200 μm. **d** Alkaline phosphatase staining of PC-iPS and PEF-iPS cells, scale bar 200 μm

PC-iPS and PEF-iPS cells were generated from PCs and PEFs, respectively. To generate PCs, the microvascular tissue was extracted from the fetal pig brain meninges and plated, which exhibited intact microvascular tube morphology; on day 3, sprouted PCs can be observed at the surrounding of microvascular tubes (Additional file 2: Figure S1 A) and the fetal PCs were spindle-shaped, proliferative, and positive for the PC-specific markers α-SMA and NG (Additional file 2: Figure S1B). PCs and PEFs were transduced with a retroviral vector encoding Oct4, Sox2, Klf4, and cMyc reprogramming factors as previously reported [13]. A mixture of mTeSR1 and a modified LCDM medium (LCDMV, see the “Methods” section for details) (1:1, *v*/*v*) was used from day 5 with medium changed every other day until passage 1 (P1); thereafter, iPS cells were cultured in LCDMV medium only. A schematic illustration of the generation of pig iPS cells in LCDMV medium is shown in Fig. 1a. After retroviral infection, the cell morphology started to change on day 4; on day 16, the PEFs formed small and compact colonies, which are different from the large and flat colonies observed in PCs (Fig. 1b). After four passages, however, both PEF- and PC-iPS cells homogenously exhibited a “dome-shaped” morphology and were competent for clonal passaging, typical features of naive PS cells (Fig. 1c). Both PC-iPS and PEF-iPS cells were also positive for alkaline phosphatase (AP) staining (Fig. 1d). In total, we generated 6 PEF-iPS and 3 PC-iPS cell lines. Primary cells of PEFs and PCs were isolated from the same pregnant Nongda xiang pig, but different fetus; PEFs were from one male fetus, but PCs were from one female fetus. Collectively, PCs and PEFs can be reprogrammed by a retroviral vector encoding Oct4, Sox2, Klf4, and cMyc to naïve-like iPS cells, which could be stably maintained in a modified LCDM culture medium (LCDMV).

### Characterization of PC-iPS and PEF-iPS cells cultured in LCDMV medium

**Fig. 2:**
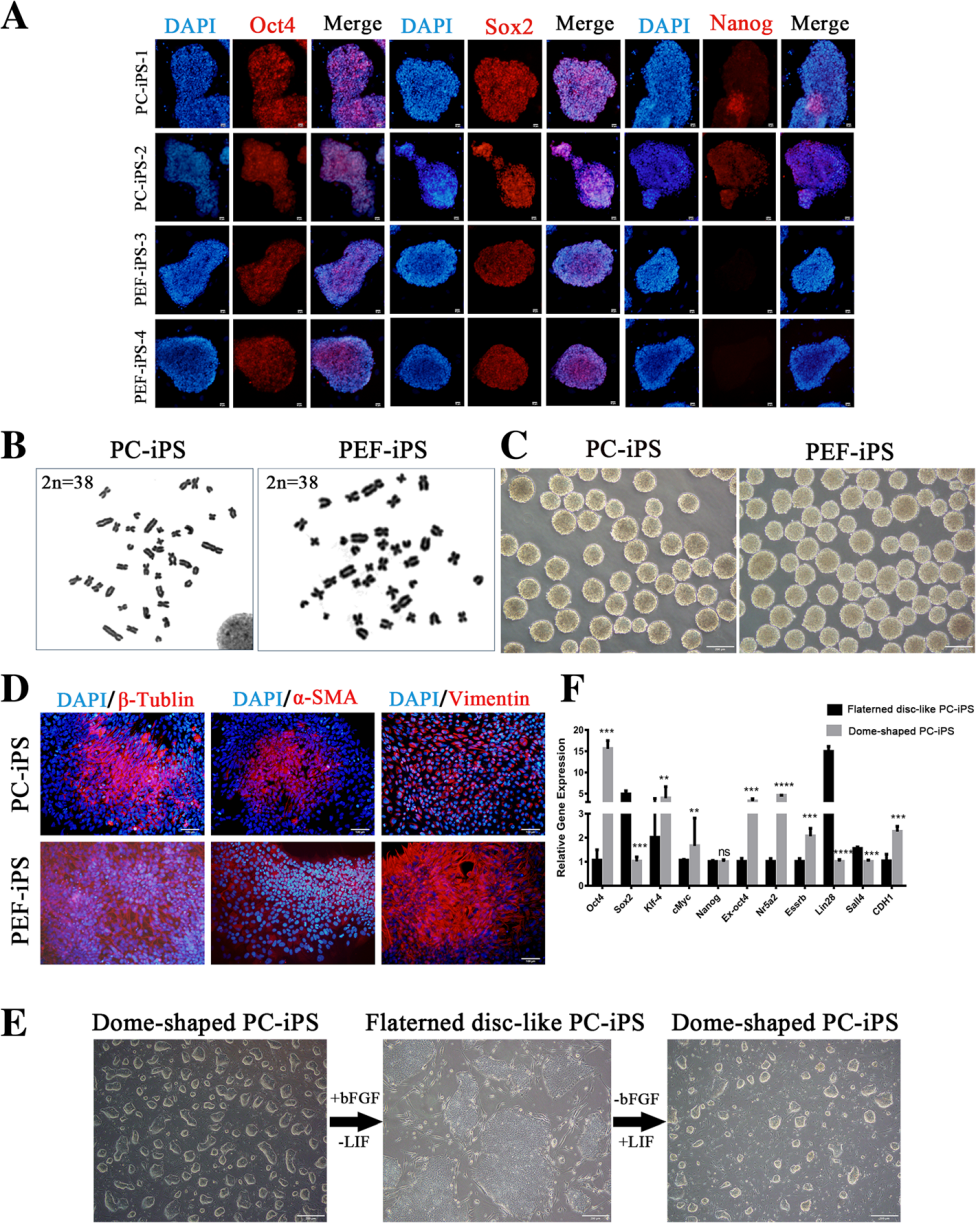
Characterization of PC-iPS and PEF-iPS cells**. a** Immunocytochemistry analysis of PC-iPS and PEF-iPS cells, scale bar 10 μm. **b** Karyotype analysis of PC-iPS and PEF-iPS cells. **c** EB formation of PC-iPS and PEF-iPS cells, scale bar 200 μm. **d** Immunohistochemistry of 3 germ layers differentiation of PC-iPS and PEF-iPS cells in vitro, scale bar 100 μm. **e** Toggling between dome- and flattened disc-shaped PC-iPS cells, scale bar 200 μm. **f** RT-PCR analysis of dome- and flattened disc-shaped PC-iPS cells (****p < 0.0001, ***p < 0.001, **p < 0.5; ns, not significant)

The immunocytochemical staining showed that both PC-iPS (P12) and PEF-iPS(P12) cells expressed OCT4, SOX2 (Fig. 2a), and SALL4 (Additional file 3: Figure S2), but only PC-iPS cells expressed NANOG (Fig. 2a) and TRA-1-81(faint signal) (Additional file 3: Figure S2). Karyotype analysis of long-term cultured PC-iPS (P28) and PEF-iPS (P18) cells were confirmed a normal diploid chromosome content in both cell types (Fig. 2b). Both PC-iPS and PEF-iPS cells formed embryonic bodies (EBs) (Fig. 2c) and generated cell derivatives of ectodermal (β-TUBULIN), mesodermal (α-SMA), and endodermal (VIMENTIN) origins (Fig. 2d)*.* Next, we tested whether PC-iPS cells could toggle between dome and flattened shape colony morphologies characteristic of naïve and primed pluripotent states, respectively. To this end, we replaced LIF with 10 ng/ml of basic fibroblast growth factor (bFGF) in LCDMV medium. After one passage, we observed the morphology of PC-iPS cell colonies changed from dome-shaped into flattened morphology. Interestingly, when LIF was added back to replace bFGF, PC-iPS colonies regained the dome-shaped morphology (Fig. 2e). Quantitative RT-PCR (qPCR) analysis showed that some pluripotency-related genes, e.g., *Oct4*, *Klf4*, *cMyc*, *Nr5a2*, and *Esrrb*, were downregulated during dome to flattened transition, but *Sox2*, *Lin28*, and *Sall4* were upregulated and Nanog remained at the same level (Fig. 2f).

### Transcriptome analysis of PC-iPS and PEF-iPS cells cultured in LCDMV medium

**Fig. 3:**
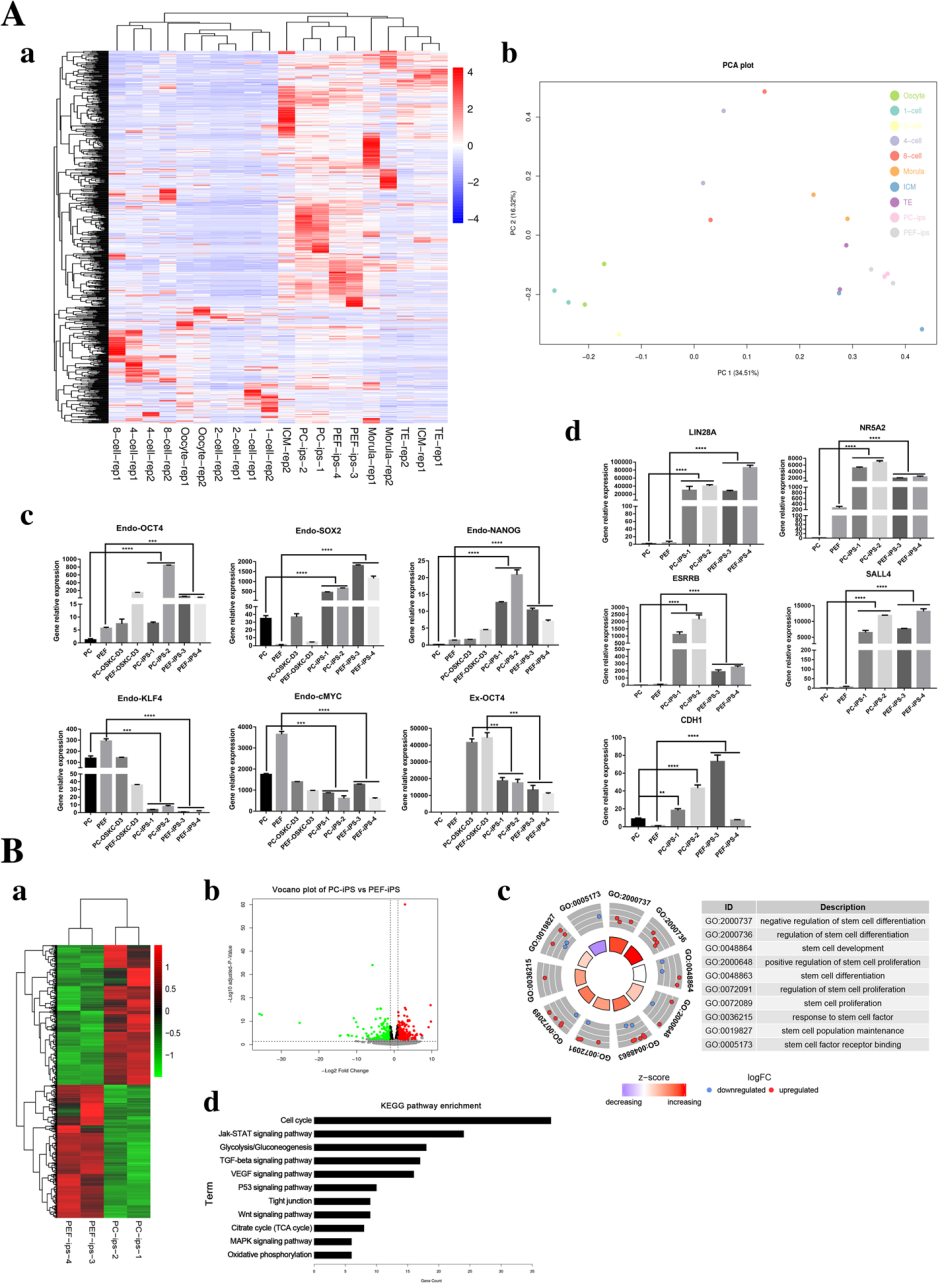
Transcriptome analysis of PEF-iPS and PC-iPS. (A, a) Heatmap analysis of PC-iPS cells, PEF-iPS cells, and different stages of pig embryos. (A, b) PCA analysis of PC-iPS cells, PEF-iPS cells, and different stages of pig embryos. (A, c) Expression analysis of endogenous and exogenous pluripotency-related transcription factors in PC-iPS and PEF-iPS cells cultured in LCDMV. (A, d) Expression analysis of other pluripotency-related genes in PC-iPS and PEF-iPS cells. (B, a) Heatmap analysis of PC-iPS vs PEF-iPS cells (*P* < 0.01). (B, b) Volcano plot of PC-iPS vs PEF-iPS cells. (B, c) GO terms of PC-iPS vs PEF-iPS cells. (B, d) KEGG pathway enrichment of signal pathways (****p < 0.0001, ***p < 0.001, **p < 0.5; ns, not significant)

Heatmap (Fig. 3A, a) and principal component analyses (PCA) (Fig. 3A, b) revealed that global transcriptional profiles of PC-iPS and PEF-iPS cells clustered closer with ICM isolated from pig blastocysts, and more distant from 1-cell to morula pig embryos. qPCR analysis showed that the expression levels of core endogenous pluripotency-related transcription factors including *Endo-Oct4*, *Endo-Sox2*, and *Endo-Nanog* were significantly upregulated in PC-iPS and PEF-iPS cells cultured in LCDMV, and exogenous *Oct4 (Ex-Oct4)* expression was downregulated (Fig. 3A, c). *Endo-KLF4* and *Endo-cMyc*, however, were at similar expression levels to the starting cells (Fig. 3A, c). Other pluripotency-related genes such as *NR5A2*, *ESRRB*, *LIN28*, *SALL4*, and *CDH1* were also upregulated in both PC-iPS and PEF-iPS cells (Fig. 3A, d). We further analyzed differentially expressed genes (DEGs) between PEF-iPS and PC-iPS cells. Heatmap (Fig. 3B, a) and volcano plot (Fig. 3B, b) showed that there were 1475 DEGs between PC-iPS and PEF-iPS cells (adjusted *p* value < 0.1) and the numbers of genes upregulated and downregulated in PC-iPS cells were 755 and 720, respectively (Additional file 4: Table S2; Additional file 5: Table S3). We performed GO and KEGG pathway enrichment analyses using the list of genes upregulated in PC-iPS cells and found that enriched GO terms (Additional file 6: Table S4) included regulation of stem cell differentiation, proliferation, development, and maintenance (Fig. 3B, c) and enriched KEGG pathways (Additional file 7: Table S5) included *Jak-STAT*, *TGF-β*, *P53*, *Wnt*, and *MAPK* stem cell signaling pathways (Fig. 3B, d). In sum, PC-iPS and PEF-iPS cells were clustered closer with pig ICMs and expressed pluripotency-related genes; analysis of DEGs suggests that PC-iPS cells express more pluripotency-related genes than PEF-iPS cells, suggestive of better iPS cell quality. Therefore, we chose PC-iPS cells for subsequent evaluation of developmental potential in vivo*.*

### Chimeric contribution of pig PC-iPS cells to mouse embryonic and extraembryonic tissues

**Fig. 4:**
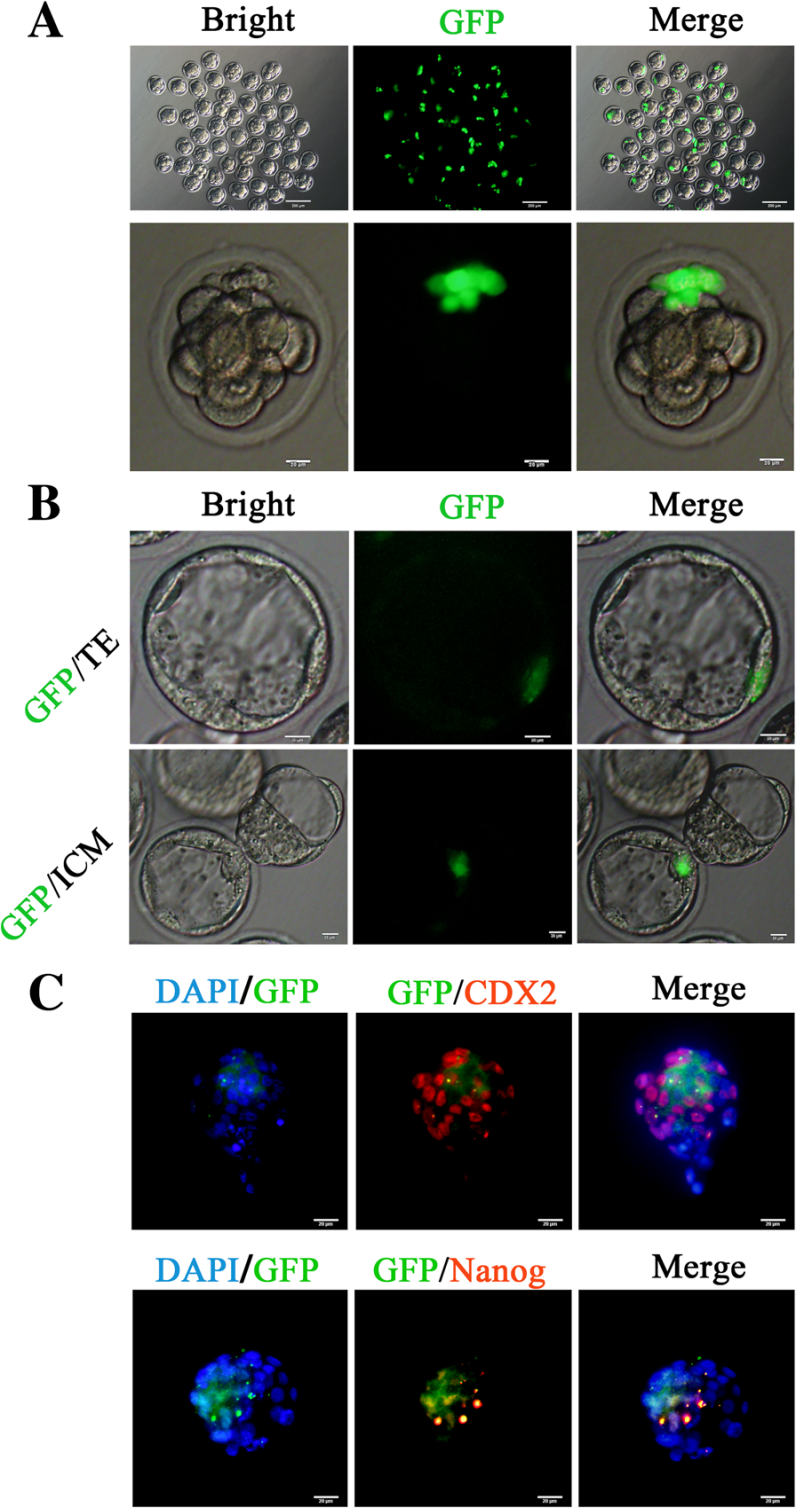
PC-iPS contributes to mouse late blastocyst TE and ICM in vitro**. a** Injection GFP labeled PC-iPS cells to 4- to 8-cell embryos, scale bar 200 μm, 20 μm. **b** GFP-labeled PC-iPS cells contribute to the TE and ICM, scale bar 20 μm. **c** Immunocytochemistry analysis of CDX2 and NANOG in chimeric mouse late blastocyst, scale bar 20 μm

To examine the developmental potential of pig PC-iPS cells cultured in LCDMV medium, we injected five to ten GFP-labeled PC-iPS cells (Additional file 8: Figure S3 A) into 4- to 8-cell mouse embryos (Fig. 4a) and examined their chimeric contribution in late blastocysts. Notably, GFP-labeled PC-iPS cells could be detected in both trophectoderm (TE) and ICM after 36 h in vitro culture post-injection (Fig. 4b). Immunocytochemistry analysis confirmed that the injected PC-iPS cells contributed to both the TE (CDX2 positive) and ICM (NANOG positive) in chimeric blastocysts (Fig. 4c). The bi-developmental potency was further confirmed by single-cell injection (Additional file 8: Figure S3C-D).

In addition to 4- to 8-cell embryo injection, we also performed blastocyst injection of GFP-labeled PC-iPS cells, and the results were similar to 4–8-cell embryo injections (Additional file 8: Figure S3B). Next, we transferred chimeric blastocysts, including 4- to 8-cell embryo injected (E2-CBs) and early blastocyst injected (E3.5-CBs), into recipient mice to examine chimeric contribution in post-implantation embryos in vivo. As shown in Table 1, the pregnancy rates of recipient mice transplanted with E2-CBs and E3.5-CBs were 28.75% and 46.67%, respectively. After embryo transfer, 13.33% E2-CBs survived to E10.5 to E13.5 stages, much less than that of E3.5-CBs at 35.27%. Among transferred chimeric blastocysts, 19.92% E3.5-CBs and 3.33% E2-CBs developed into normal sized fetuses. Among all implanted embryos, the rate of normal sized fetuses is 25% for E2-CBs and 56.47% for E3.5-CBs. In sum, injecting pig iPS cells into early mouse blastocysts resulted in better developmental outcomes than 4- to 8-cell embryo injection.

**Table 1:** Comparison of the outcomes between 4- to 8-cell embryo injection and blastocyst injection

Chimera type	Total no. of chimera embryos transplanted into recipient mice	Total no. of recipient mice	Total no. of pregnant mice	Total no. of normal fetuses recovered	Total no. of other recovered (implantation site without a definable embryo, necrotic or reabsorbing implantation)	Total no. of pregnant mice/total no. of recipient mice (%)	Total no. of normal fetus recovered/total no. of chimera embryos transferred (%)	(Total no. of normal fetuses + total no. of other recovered)/total no. of chimera embryos transplanted (%)	Total no. of normal fetus recovered/(total no. of normal recovered + total no. of others recovered) (%)
Mouse 4- to 8-cell embryo injection	240	14	4	8	24	4/14 (28.57%)	8/240 (3.33%)	(8 + 24)/240 (13.33%)	8/(8 + 24) (25%)
Mouse blastocyst injection	241	15	7	48	37	7/15 (46.67%)	48/241 (19.92%)	(48 + 37)/241 (35.27%)	48/(48 + 37) (56.47%)

**Fig. 5:**
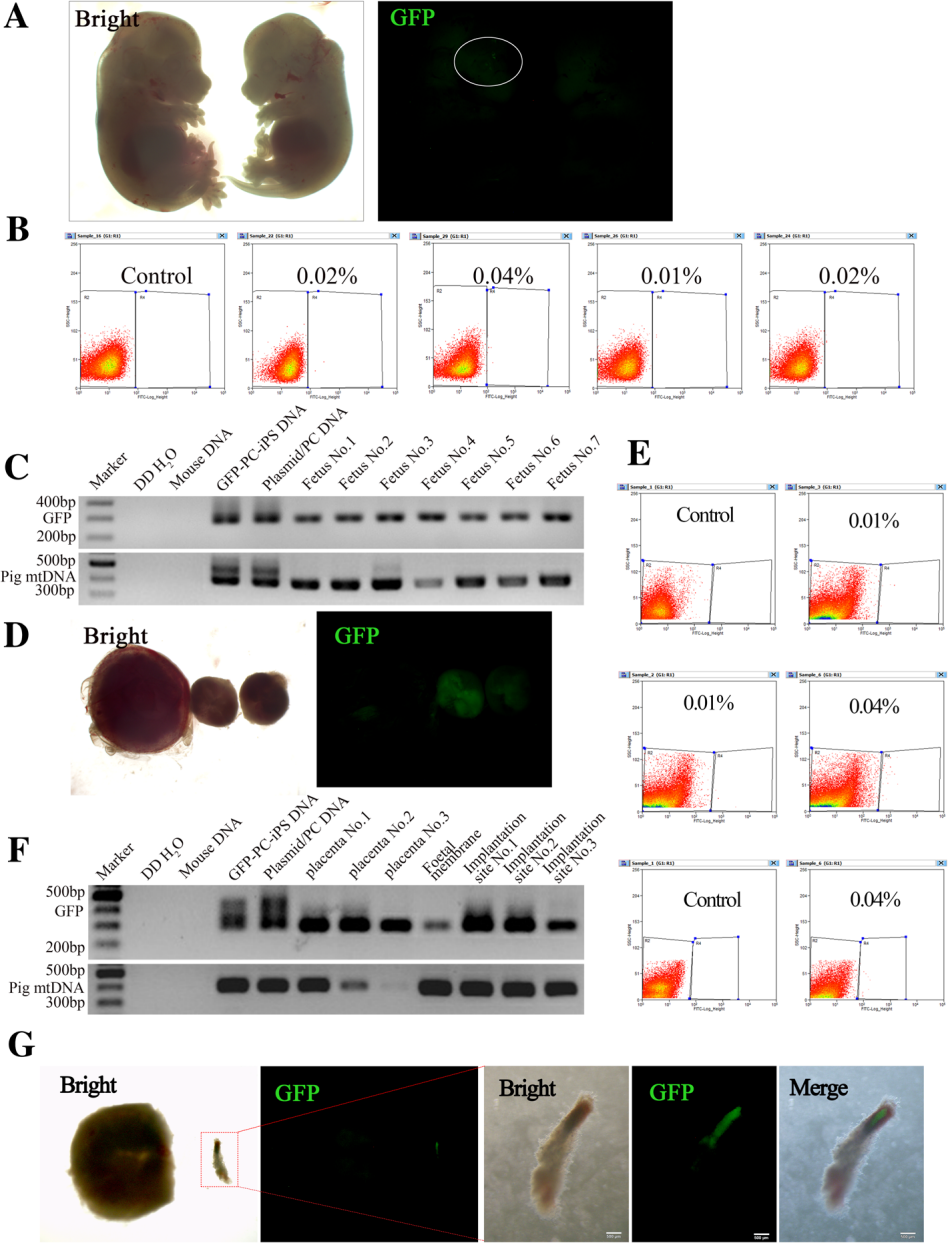
Analysis GFP PC-iPS contribution to chimera mouse in vivo***.*** **a** Representative bright-field and fluorescence images of chimeric mouse fetuses. **b** Flow cytometry analysis of GFP positive cells of chimeric mouse fetuses. **c** Nested PCR of GFP and pig mtDNA of chimeric mouse fetuses (the GFP-labeled PC-iPS cells’ DNA and PB513B-1 plasmid were used as GFP sequence positive controls; the GFP-labeled PC-iPS cells’ DNA and PC DNA were used as pig mtDNA sequence positive control; double distilled water (ddH_2_O) and mouse DNA were used as negative controls). **d** Representative bright-field and fluorescence images of extraembryonic tissues. **e** Flow cytometry analysis of GFP-positive cells within the extraembryonic tissue. **f** Nested PCR of GFP and pig mtDNA of chimeric mouse extraembryonic tissues. **g** Representative bright-field and fluorescence images of degenerated mouse embryo, scale bar 500 μm

To check post-implantation chimeric contribution of pig PC-iPS cells, mouse conceptuses between E10.5 and E13.5 stages were collected and analyzed. As shown in Fig. 5a, GFP signal could be detected in the head of a fetus and the level of chimeric contribution was found up to 0.04% (Fig. 5b). To confirm chimeric contribution, nested PCR was used to detect the presence of pig mtDNA-specific and the GFP sequence. The GFP-labeled PC-iPS cells’ DNA and PB513B-1 plasmid were used as GFP sequence positive controls; the GFP-labeled PC-iPS cells’ DNA and PC DNA were used as pig mtDNA sequence positive control; double distilled water (ddH_2_O) and mouse DNA were used as negative controls. As the nested PCR data shown, pig mtDNA could be detected in the fetus, confirming the chimeric contribution of pig PC-iPS cells (Fig. 5c). We also checked whether pig PC-iPS cells could contribute to mouse extraembryonic tissues. To this end, we isolated placentas, amniotic membranes, and implantation sites for analysis. The GFP signals could also be detected in some extraembryonic tissues (Fig. 5d), and the level of chimeric contribution to extraembryonic tissue was found up to 0.04% (Fig. 5e). The chimerism was further confirmed by PCR using primers for GFP and pig mtDNAs (Fig. 5f). Moreover, we found some of the degenerated embryos were strongly positive for GFP (Fig. 5g). Taken together, PC-iPS cells could contribute to chimeric formation in ICM and TE of pre-implantation mouse blastocysts, and in embryonic and extraembryonic tissues, at a very low level, of post-implantation mouse conceptuses in vivo*.*

### Chimeric contribution of pig PC-iPS cells to pig embryos

**Fig. 6:**
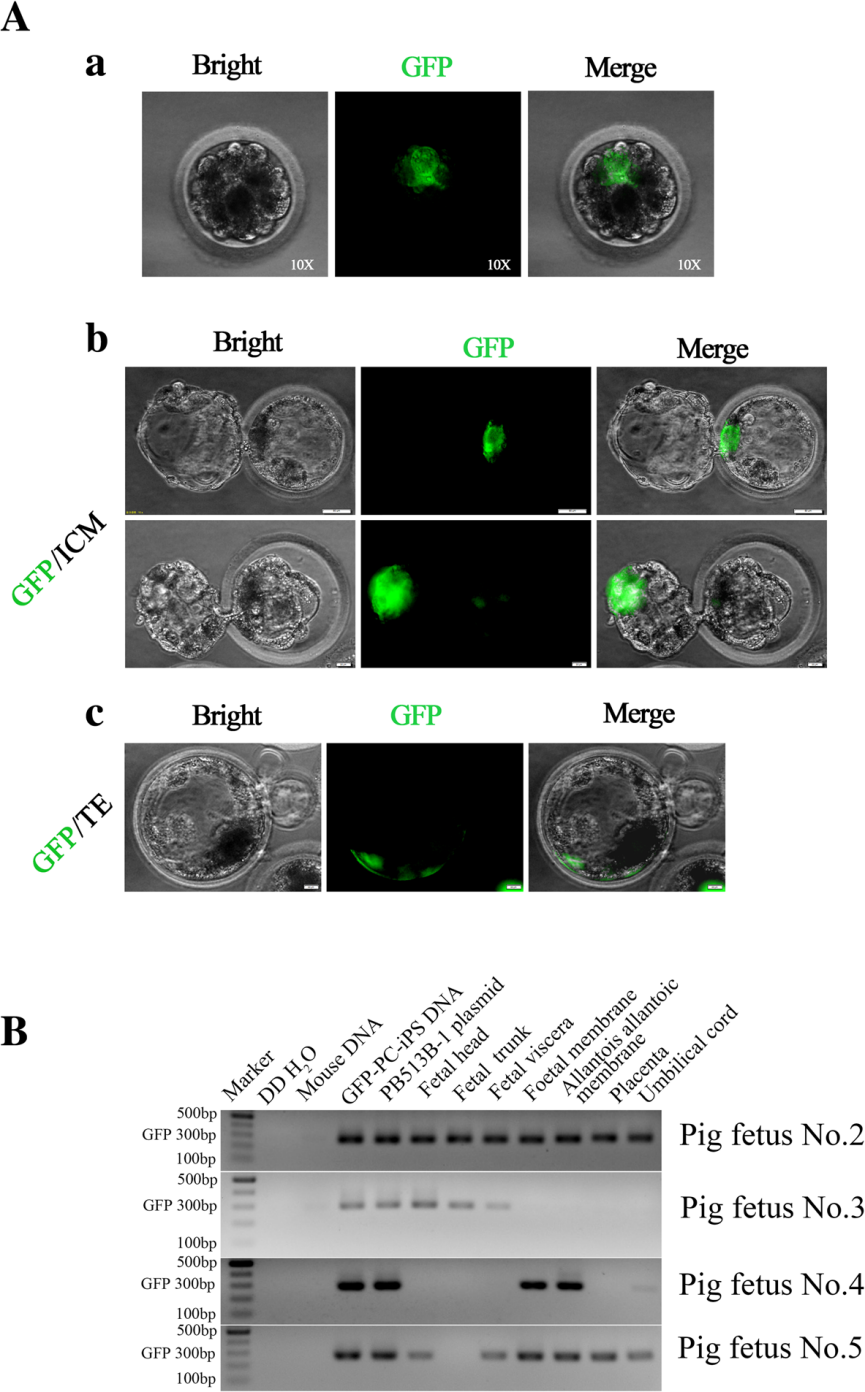
GFP-labeled PC-iPS cells contribute to chimeric formation in pig embryos in vitro and in vivo**.** (A, a) Representative fluorescence images of pig embryo injected with GFP-labeled PC-iPS cells, scale bar 10 μm. (A, b) GFP-labeled PC-iPS contribute to the pig ICM, scale bar 50 μm. (A, c) GFP-labeled PC-iPS cells contribute to the pig TE, scale bar 50 μm. (B) Nested PCR analysis of GFP using post-implantation pig fetal embryonic and extraembryonic tissues (the GFP-labeled PC-iPS cells’ DNA and PB513B-1 plasmid were used as GFP sequence positive controls; double distilled water (ddH_2_O) and mouse DNA were used as negative controls)

Next, to examine whether PC-iPS cells could participate in normal pig development, we injected ten to 15 GFP-labeled PC-iPS cells into SCNT-derived porcine 4- to 8-cell embryos (Fig. 6A, a). When cultured to blastocysts, GFP-labeled PC-iPS cells could be detected in the ICM (Fig. 6A, b) and TE (Fig. 6A, c). Next, a total of 1673 injected SCNT embryos was transferred into 7 surrogate sows, which gave rise to 4 live fetuses between E25 and E30 (Table 2). Embryonic and extraembryonic tissues from the 4 fetuses were isolated and subjected to further analysis for chimeric contribution. Although we did not detect any GFP signal in all 4 pig conceptuses under a fluorescent stereoscope, nested genomic PCR revealed that GFP sequence could be detected in embryonic (head, trunk, and viscera) and extraembryonic tissues (fetal membrane, allantois, placenta, and umbilical cord) examined in one pig conceptus (No. 2), and the other 3 conceptuses contained GFP sequence in some tissues (Fig. 6B). Overall, PC-iPS cells could contribute to chimeric formation in both ICM and TE of pig blastocysts, as well as embryonic and extraembryonic tissues, at a very low level, of post-implantation pig conceptuses*.*

**Table 2:** Summary of the outcomes of injected pig embryos transferred to recipient sows

Recipient pig ID	No. of GFP-PC-iPS chimera embryos transferred to recipient pig	Result of type-B ultrasonic scanning (pregnancy Y/N)	Total fetuses collected (*N*, no fetus collected)
H304	200	N	N
H110	150	Y	N
H374	222	Y	N
H350	250	Y	N
H329	214	Y	4
H312	207	N	N
H337	430	N	N
Total	1673		4

## Discussion

In this study, a mixture of 50% mTeSR1 and 50% LCDMV media (*v*/*v*) was used during PCs and PEFs reprogramming. On day 16, PC-iPS cells formed large and flat colonies, while PEF-iPS cells formed small and dome-shaped colonies. mTeSR1 is a widely used commercial human primed pluripotent culture medium, and primed human ES/iPS cells are dependent on FGF [32] and Nodal/Activin A [33] signal pathways to maintain pluripotency. It has been reported that genes involved in FGF signaling pathways are expressed in D5/6 pig blastocysts [34] and pig iPS cells can be stably maintained in the primed pluripotent state by using bFGF containing medium [13]. It is likely that the addition of mTeSR1 culture medium strengthens FGF and/or Nodal/Activin A signal pathway(s) that are important for the initial phase of reprogramming. The cell proliferation at the first phase of reprogramming is crucial for successful reprogramming. bFGF and TGF-β can promote cell proliferation. Thus, mTeSR1 containing bFGF and TGF-β can facilitate reprogramming. Our results also demonstrate that PC-iPS cells could easily switch between dome-shaped and flattened colony morphologies, which are characteristics of naïve and primed pluripotent states, respectively, by simply toggling between bFGF and LIF in the culture medium. These observations highlight an active role of the FGF signaling pathway during cellular reprogramming and maintenance of pluripotency in pig iPS cells.

Germline chimeras are widely accepted as the gold standard for assessing the pluripotency of ES or iPS cells [35]. In this study, we showed that PC-iPS cells cultured in LCDMV medium could contribute to chimeric formation in post-implantation pig conceptuses. Future studies are warranted to examine whether PC-iPS cells can contribute to germline chimeras. Interspecies chimeras have also been employed to assess the pluripotency of human ES or iPS cells [36, 37], and the successful generation of human-pig chimeric embryos marked the first step toward solving the worldwide shortage of organ donors in the future [38]. We also examined chimeric contribution of pig PC-iPS cells to mouse. Our results show that PC-iPS cells could differentiate and contribute to TE and ICM of pre-implantation mouse blastocysts. Following embryo transfer, some of the mouse conceptuses contained pig PC-iPS cell derivatives in both embryonic and extraembryonic tissues. Flow cytometry analysis showed that the levels of chimerism were up to 0.04%. This is in line with several recent studies of human-mouse [39], human-pig and mouse/rat-pig chimeras [38], which demonstrated that the level of interspecies chimerism is low, which may reflect the xenogeneic barrier between evolutionary distant species.

In addition to LCDM medium, another medium termed EPSCM [40] also supported the generation of EPS cells from mouse embryos that display bi-developmental potency toward both embryonic and extraembryonic lineages. To date, pig EPS cells using either culture condition have not been derived from in vivo embryos. In the current study, we succeeded in the generation of pig iPS cells using a modified version of LCDM medium and these cells showed the bi-developmental potency characteristic of mouse and human EPS cells. However, the level of chimerism in pig PC-iPS was very low. There are several possible explanations: (1) exogenous genes used for reprogramming were found not silenced in PC-iPS cells, similar to other culture conditions used [12], which might have affected proper differentiation of pig PC-iPS cells in mouse or pig conceptuses. Thus, it will be imperative to test de novo derivation of pig EPS cells from pig blastocysts and examine their chimeric contribution in pigs and mice. (2) Signaling pathways maintaining the pluripotency program in pig embryos likely differ from mouse and human. Activation of LIF-JAK/STAT3 pathway is important to maintain pluripotency program in mouse naïve ES/iPS cells [41]. LIF and LIFR are expressed in mouse ICM [42] and human ICM [43], but not in pig ICM and epiblast [34, 44]. Therefore, LIF signaling pathway may not play an active role in pig embryo development. Interestingly, pig ICM highly expressed IL6ST and IL-6 receptor (IL6R) [45], and whether they play key roles in pig pluripotency program remains unknown. Inhibition of Erk and MEK (2i) pathways enabled efficient derivation of murine naive ES/iPS cells [5, 46], but does not work for pigs [47]. PARP1 and histamine and muscarinic receptor signaling inhibition play an important role in maintaining mouse EPS developmental potency [16], and whether these pathways play similar roles in pig ES/iPS cells needs to be further studied. (3) Pig pluripotency program may differ from mouse and human. Recently, single-cell RNA-seq analysis of different stages of pig embryos showed that naïve pluripotent genes *(KLF4*, *KLF5*, *KLF17*, *TFCP2L1*, *ESRRB*, *TBX3)* and prime pluripotent genes *(PRDM14*, *NODAL*, *DNMT3B*, *SALL2*, *SFRP2*, *FGF2*, *SOX11)* [45] in pigs are different from mouse [48], human [49, 50], and monkey [51].

## Conclusions

Taken together, our current study reports the successful generation of stable naïve-like pig iPS cells using a modified EPS culture (LCDMV). Derived pig iPS cells could differentiate into cells representative of three germ lineages in vitro and showed chimeric contribution to both embryonic and extraembryonic tissues in post-implantation pig conceptuses. Upon further optimization, LCDMV culture may support de novo derivation of germline-competent pig EPS cells from early embryos.

## Additional files

Additional file 1: **Table S1.** List of primers used in this study. (PDF 122 kb)

Additional file 2: **Figure S1.** Morphology and immunocytochemistry analysis of pig meninges pericytes (PCs). **(**A) Microvascular tubes attached, and PC sprouted from day 0 to day 3, and morphology of PCs at passage 3. Scale bar 100 μm. (B) PCs stained positive for α-SMA and NG2, scale bar 200 μm. (PNG 6480 kb)

Additional file 3: **Figure S2.** Immunocytochemistry analysis of PC-iPS and PEF-iPS cells, scale bar 20 μm. (PNG 2001 kb)

Additional file 4: **Table S2.** The expression levels (RPKM) from RNAseq analysis of PC-iPS and PEF-iPS cells. (XLS 1109 kb)

Additional file 5: **Table S3.** Differential expression genes between PC-iPS and PEF-iPS cells. (XLS 2135 kb)

Additional file 6: **Table S4.** GO enrichment of differentially expressed genes. (XLS 1166 kb)

Additional file 7: **Table S5.** KEGG enrichment of differentially expressed genes. (XLS 25 kb)

Additional file 8: **Figure S3.** blastocysts injection of GFP-labeled PC-iPS and single-cell injection. (A) Labeling PC-iPS with GFP, scale bar 50 μm; (B) blastocyst injection of GFP-labeled PC-iPS cells, scale bar 50 μm, 10 μm; (C) single GFP PC-iPS cell injection, scale bar 200 μm, scale bar 20 μm; (D) single GFP PC-iPS cell contribution to ICM and TE respectively, scale bar 10 μm. (PNG 8812 kb)

## Data Availability

The datasets generated during and/or analyzed during the current study are available from the corresponding author on reasonable request. All data generated or analyzed during this study are included in this published article (and its supplementary information files). The datasets generated during and/or analyzed during the current study are not publicly available due to reason(s) why data are not public but are available from the corresponding author on reasonable request.

## Abbreviations

AP: Alkaline phosphatase
bFGF: Basic fibroblast growth factor
CHIR: CHIR99021
DIM: (S)-(+)-Dimethindene maleate
E2-CBs: 4–8 cells stage injected pig-mouse chimeric blastocysts
E3.5-CBs: Blastocyst stage injected pig-mouse chimeric blastocysts
Endo genes: Endogenous genes
EPS: Expanded pluripotent stem cells
ES: Embryonic stem cell
Ex genes: Exogenous genes
GFP: Green fluorescence protein
hCG: Human chorionic gonadotropin
hLIF: Human leukemia inhibitory factor
ICM: Inner cell mass
iPS: Induced pluripotent stem
KOSR: Knockout serum replacement
LCDM: LCDMV without vitamin C
LCDMV: 50% (*v*/*v*) Neurobasal™ Medium, 50% (*v*/*v*) DMEM/F12, N2 (100×), B27 (50×), 0.5% KOSR, LIF (10 ng/ml), CHIR9 (1 μM), (S)-(+)-dimethindene maleate (2 μM), minocycline hydrochloride (2 μM), and vitamin C (40 μg/ml)
MEFs: Mouse embryonic fibroblasts
MIH: Minocycline hydrochloride
PC-iPS: Induced pluripotent stem cells derived from PCs
PCs: Pig meninges pericytes
PEF- iPS (2i): Cultured PEF-iPS with 2 inhibitors (MERK inhibitor and ERK inhibitor)
PEF-iPS: Induced pluripotent stem cells derived from PEFs
PEFs: Pig embryonic fibroblasts
Pig mtDNA: Pig mitochondrial DNA
PMSG: Regnant mare serum gonadotropin
SCNT: Somatic nuclear transfer
TE: Trophectoderm
