# Supplementary material for: Generation of pig induced pluripotent stem cells using an extended pluripotent stem cell culture system
Source: Stem Cell Res Ther. 2019 Jun 27;10:193. doi: 10.1186/s13287-019-1303-0 (PMC6598264; doi:10.1186/s13287-019-1303-0)
Supplement: Supplementary file 1 — Table S1. List of primers used in this study. (PDF 122 kb) [file 13287_2019_1303_MOESM1_ESM.pdf]

Additional file 1:Table 1. Primer list

Gene name	Primer
**Endo-Oct4**	F: CTTCACCACCCTGTACTCCTC
	R: GCTTCTCTCCCTAGCTCACC
**Endo-Sox2**	F: CATCAACGGTACACTGCCTCTC
	R: ACTCTCCTCCCATTTCCCTCTTT
**Endo-Klf4**	F: GAGGGAAGACCAGAATCCCTTGTA
	R: TAGAACCAAGACTCACCAAGCACC
**Endo-cMyc**	F: CACAGCTTACATCCTGTCTGTCCA
	R: GCCATTCTAGTTCCTCCCTCCAAT
**Endo-Nanog**	F: CCTACAATCCAGCTCTTTGG
	R: CTCAGGCATTGGTGAAGATT
**Ex-Oct4**	F: GACGGCATCGCAGCAGCTTGGATACAC
	R: GAGAAGGCGAAGTCGGAAG
**Nested GFP first PCR**	F: CCGCATCGAGAAGTACGAGG
	R: GCAGAATTGGACGACTGAGC
**Nested GFP second PCR**	F: TCACCGACAAGATCATCCGC
	R: TTGAAGGCGTGCTGGTACTC
**Nested pig mtDNA first PCR**	F: GACCTCCCAGCCCCCTCAAACATCTCATCATGATGAAA
	R: GCTGATAGTAGATTTGTGATGACCGTA
**Nested pig mtDNA second PCR**	F: ACCTTAATATGACCAACATCCGA
	R: GGTAATGATGAATGGCAGGATAAAG
**EF1α**	F: AATGCGGTGGGATCGACAAA
	R: CACGCTCACGTTCAGCCTTT
**Nr5a2**	F: CACAGTTCCGAGTCGTTTCA
	R: CACTTTGCTATCTTTCCAGGC
**Essrb**	F: ACAAACTCTTCCTGGAGATGC
	R: GGACTTGAATGTTGAGGCTG
**CDH1**	F:GCAATCACCTCACGGGAAT
	R: TTATCAGCACCCACGCAA
**Lin28a**	F: GTTCTGCATTGGGAGCGAGA
	R: GGCAGTTTGCATTCCTTGGC
**Sall4**	F: GCTGTGCCGAGTTCTTCAGT
	R: TCGCTGTGAGTGTCCTTCCT
