# Supplementary figures and images for: Generation of pig induced pluripotent stem cells using an extended pluripotent stem cell culture system
Source: Stem Cell Res Ther. 2019 Jun 27;10:193. doi: 10.1186/s13287-019-1303-0 (PMC6598264; doi:10.1186/s13287-019-1303-0)

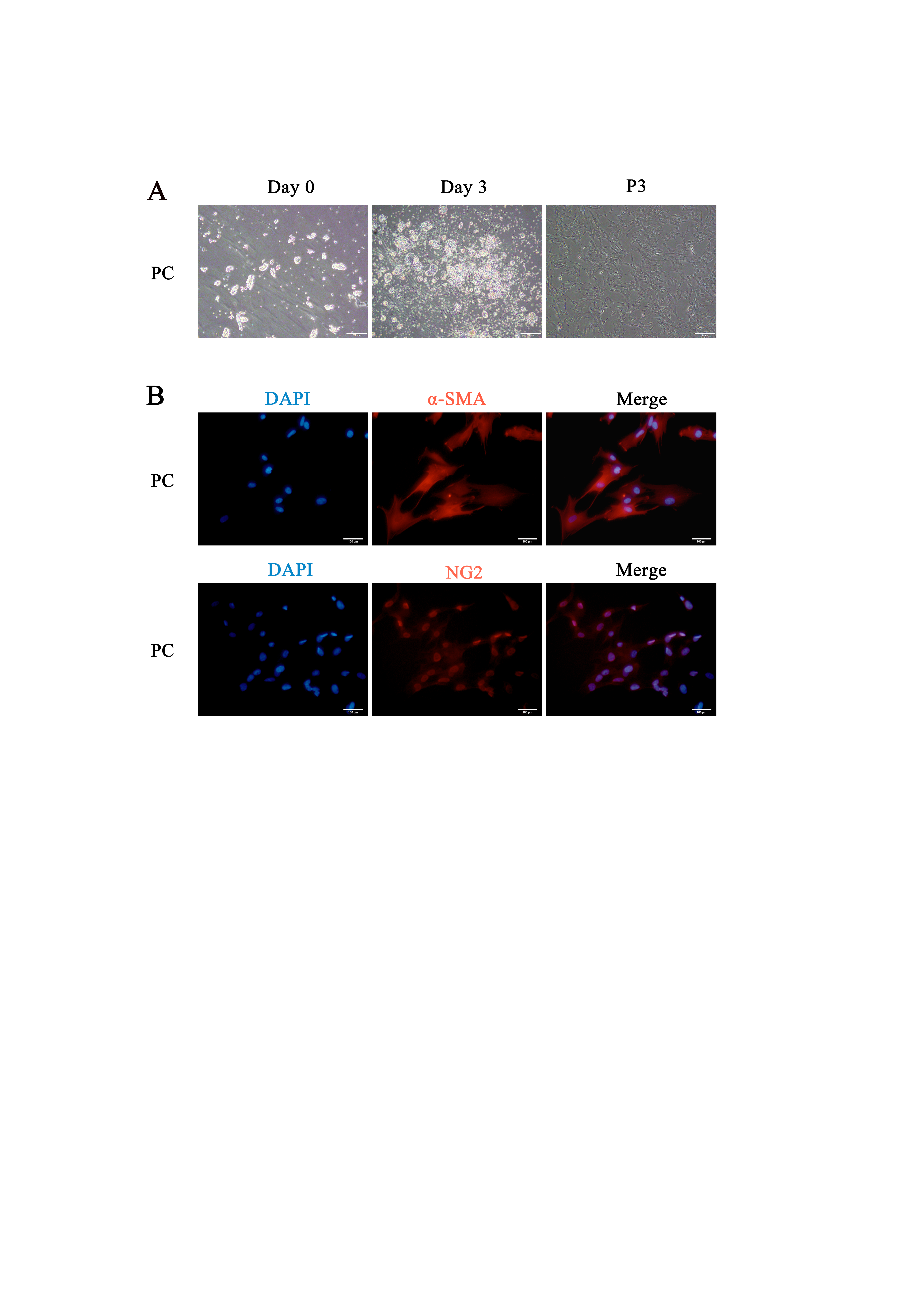

Supplement: Supplementary file 2 — Figure S1. Morphology and immunocytochemistry analysis of pig meninges pericytes (PCs). (A) Microvascular tubes attached, and PC sprouted from day 0 to day 3, and morphology of PCs at passage 3. Scale bar 100 μm. (B) PCs stained positive for α-SMA and NG2, scale bar 200 μm. (PNG 6480 kb) [file 13287_2019_1303_MOESM2_ESM.png]

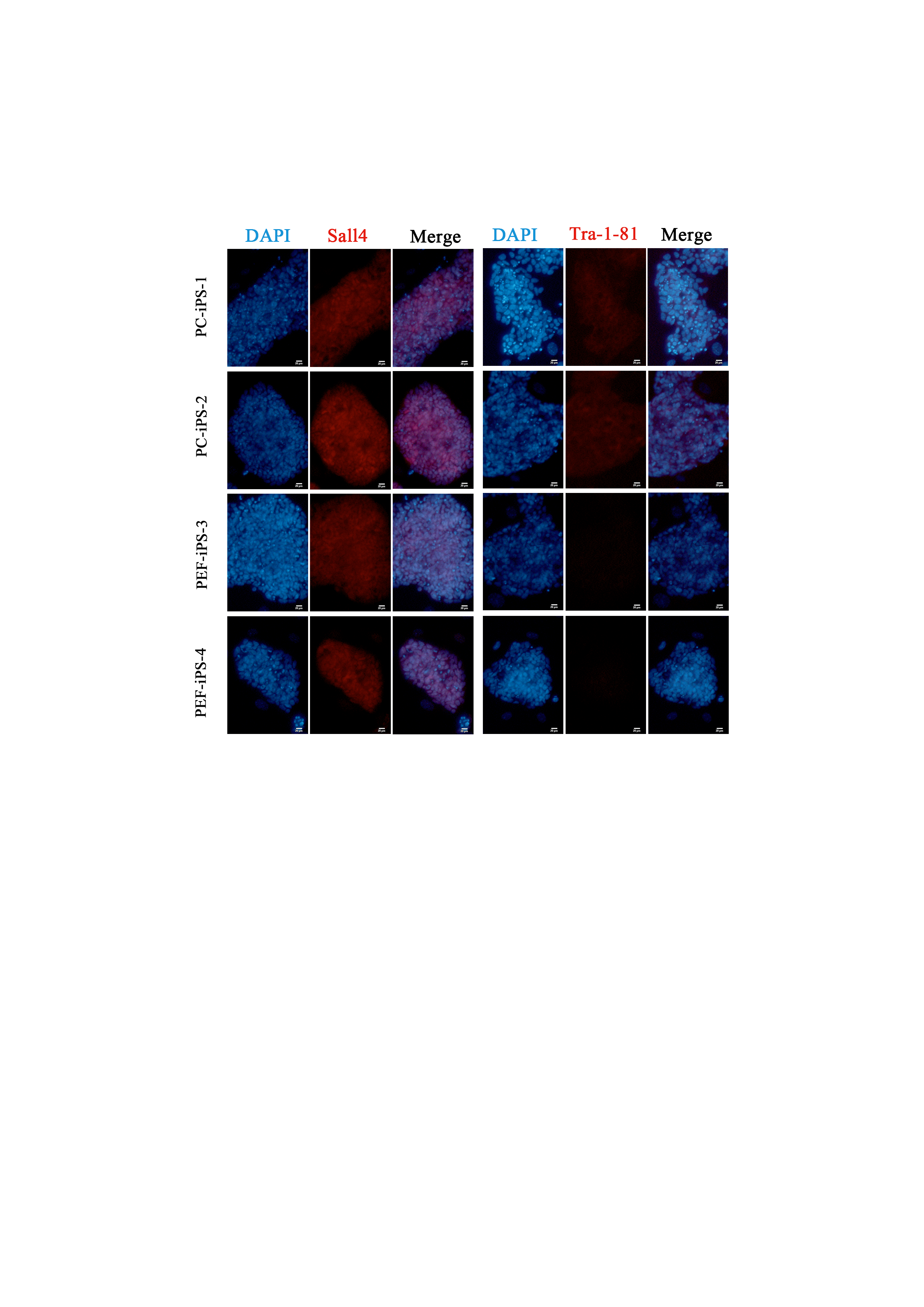

Supplement: Supplementary file 3 — Figure S2. Immunocytochemistry analysis of PC-iPS and PEF-iPS cells, scale bar 20 μm. (PNG 2001 kb) [file 13287_2019_1303_MOESM3_ESM.png]

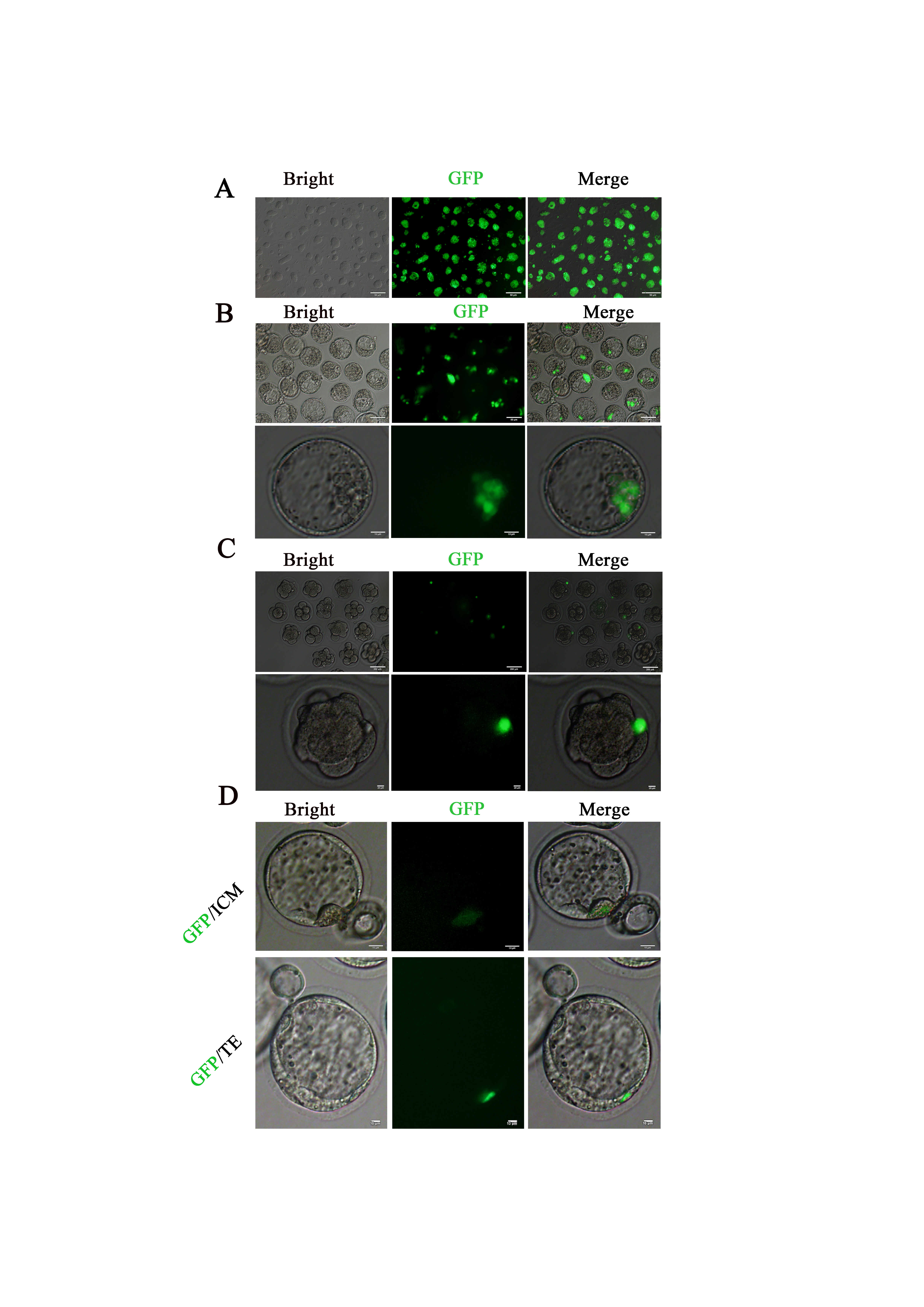

Supplement: Supplementary file 8 — Figure S3. blastocysts injection of GFP-labeled PC-iPS and single-cell injection. (A) Labeling PC-iPS with GFP, scale bar 50 μm; (B) blastocyst injection of GFP-labeled PC-iPS cells, scale bar 50 μm, 10 μm; (C) single GFP PC-iPS cell injection, scale bar 200 μm, scale bar 20 μm; (D) single GFP PC-iPS cell contribution to ICM and TE respectively, scale bar 10 μm. (PNG 8812 kb) [file 13287_2019_1303_MOESM8_ESM.png]
